# Strategies, Achievements, and Potential Challenges of Plant and Microbial Chassis in the Biosynthesis of Plant Secondary Metabolites

**DOI:** 10.3390/molecules29092106

**Published:** 2024-05-02

**Authors:** Taotao Han, Guopeng Miao

**Affiliations:** 1 Department of Bioengineering, Huainan Normal University, Huainan 232038, China; hantt748@nenu.edu.cn; 2 Key Laboratory of Bioresource and Environmental Biotechnology of Anhui Higher Education Institutes, Huainan Normal University, Huainan 232038, China

**Keywords:** plant secondary metabolites, in vitro cultivation, metabolic engineering, yeast, *E. coli*

## Abstract

Diverse secondary metabolites in plants, with their rich biological activities, have long been important sources for human medicine, food additives, pesticides, etc. However, the large-scale cultivation of host plants consumes land resources and is susceptible to pest and disease problems. Additionally, the multi-step and demanding nature of chemical synthesis adds to production costs, limiting their widespread application. In vitro cultivation and the metabolic engineering of plants have significantly enhanced the synthesis of secondary metabolites with successful industrial production cases. As synthetic biology advances, more research is focusing on heterologous synthesis using microorganisms. This review provides a comprehensive comparison between these two chassis, evaluating their performance in the synthesis of various types of secondary metabolites from the perspectives of yield and strategies. It also discusses the challenges they face and offers insights into future efforts and directions.

## 1. Introduction

The origin and early diversification of land plants represent a groundbreaking period in the history of plant life. To adapt to the transition from aquatic to terrestrial environments, plants underwent various physiological adaptations, leading to the emergence of numerous secondary metabolites and metabolic pathways controlling their biosynthesis [1]. Around 1850, pharmaceutical organic chemists began to notice the functions of these secondary metabolites and estimated their structures through extraction, isolation, and spectral identification. They also attempted to reconstruct them through chemical synthesis [2]. After years of scientific exploration, secondary metabolites have become essential sources for human medicine, pesticides, fragrances, dyes, and food additives [3].

Despite the vast diversity and quantity of bioactive substances in the plant kingdom, the majority of them are present in plants at very low concentrations. Large-scale cultivation requires extensive land use, faces challenges from pests and diseases, and entails high expenses. Chemical synthesis often involves numerous steps and harsh conditions, making it a less than optimal choice. In vitro cultivation and the metabolic engineering of plants have been used for many years to enhance the synthesis of plant secondary metabolites [4]. However, with the advancements in synthetic biology, an increasing focus has shifted toward heterologous synthesis using microorganisms [5].

This review investigates the strategies, achievements, and potential challenges in the synthesis of plant secondary metabolites using both plant-based and microbial-based chassis. In the case of plant-based chassis, the emphasis is placed on the in vitro cultivation and genetic modification of natural hosts. For model plant hosts, such as *Nicotiana benthamiana*, the review of Liu et al. [6] provides guidance on their engineering and utilization. In terms of microorganisms, we primarily focus on the key hosts of synthetic biology, *E. coli* and yeast. Other non-model microorganisms can be found in the review by Liu et al. [7].

## 2. Types and Biosynthesis of Plant Secondary Metabolites

Plant secondary metabolites are diverse in type and originate from various biosynthetic pathways. Based on their biosynthetic origins, plant natural products can be categorized into three main classes: terpenoids, alkaloids, and polyphenols [8].

**Figure 1:**
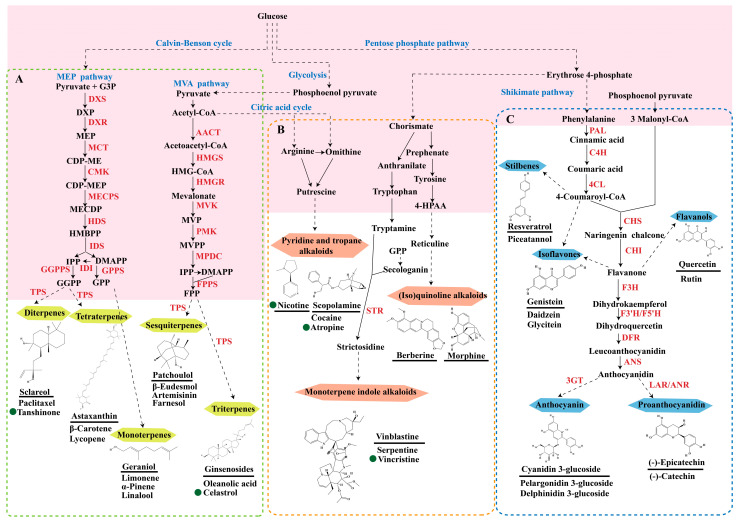
The biosynthetic pathway of terpenoids (**A**), alkaloids (**B**), and phenylpropanoids (**C**) in plants. The orange shaded area represents endogenous metabolic pathways present in microorganisms. The catalytic enzymes are shown in blue, intermediates in black. Solid arrow represents one step reaction, and dashed arrow represents multiple steps. The structures of typical natural products with underlines are displayed. Green circles indicate compounds for which the synthesis pathways have not been fully elucidated. Abbreviations: DXS,1-deoxy-D-xylulose 5-phosphate synthase; DXR, 1deoxy-D-xylulose 5-phosphate reductoisomerase; MCT, 2-C-methyl-D-erythritol 4-phosphate cytidylyltransferase; CMK,4-(cytidine 5‘diphospho)-2-Cmethyl-D-erythritol kinase; MECPS:ME-CDP synthase; HDS, 4-hydroxy-3-methylbut-2-enyl-diphosphate synthase; IDS: isoprenyl diphosphate synthase; IDI, isopentenyl diphosphate isomerase; GPP, geranyl diphosphate; GPPS, GPP synthase; GGPP, geranylgeranyl diphosphate; GGPPS, GGPP synthase; TPS: terpene synthase; AACT, acetyl-CoA acetyltransferase; HMGS, hydroxymethylglutaryl-CoA synthase; HMGR, 3-hydroxy-3-methylglutaryl-CoA reductase; MVK, mevalonate kinase; PMK, phosphomevalonate kinase; MPDC: mevalonate diphosphate decarboxylase; FPP, farnesyl diphosphate; FPPS, FPP synthase; STR: strictosidine synthase; PAL: phenylalanine ammonia lyase; C4H, cinnamate 4-hydroxylase; 4CL, 4-coumarate: CoA ligase; CHS, chalcone synthese; CHI, chalcone isomerase; F3H, flavanone 3-hydroxylase; F3′H, flavonoid-3′-hydroxylase; F5′H, flavonoid-5′-hydroxylase; DFR, dihydroflavonol reductase; ANS, Anthocyanidin synthese; 3GT: flavonoid 3-O-glucosyltransferase; LAR: Leucoanthocyanidin reductase; ANR: anthocyanidin reductase.

Terpenoids are a group of compounds that include all isoprene polymers and their derivatives. They are based on the five-carbon isoprene unit and are also known as isoprenoids. Terpenoids are categorized into acyclic (linear) and cyclic terpenoids based on the way the isoprene units are connected [9]. These compounds are synthesized by the condensation of isopentenyl diphosphate (IPP) and dimethylallyl diphosphate (DMAPP) (Figure 1A) [10]. IPP is converted into DMAPP by the action of IPP isomerase, and both IPP and DMAPP are further converted into geranyl pyrophosphate (GPP) by the action of isoprenyl transferases. Farnesyl pyrophosphate (FPP) and geranylgeranyl pyrophosphate (GGPP) serve as non-cyclic intermediates and are further transformed into various classes of terpenoid compounds through the action of terpenoid synthases [11,12]. Terpenoids are the most diverse class of secondary metabolites in plants, with highly variable chemical structures. They play crucial roles in various aspects of plant growth and development, such as in the biosynthesis of plant hormones like gibberellins, abscisic acid, strigolactones, and cytokinins. Terpenoids also contribute to the adaptation to environmental stresses, as they include compounds like allelopathic substances, defensive compounds against herbivores, and chemicals involved in allelopathy [13]. Moreover, plant terpenoids have significant medicinal and economic value, including well-known drugs like artemisinin for malaria treatment and paclitaxel as an anti-cancer agent [10].

Alkaloids are the largest class of nitrogen-containing organic compounds among secondary metabolites. They mainly include terpenoid indole alkaloids, isoquinoline alkaloids, tropane alkaloids, and pyridine alkaloids [14]. Most alkaloids are synthesized from L-amino acids (such as tryptophan, tyrosine, phenylalanine, lysine, and arginine) individually or in combination with steroids, secoiridoid (e.g., strychnoside), or other terpene ligands (Figure 1B). Terpenoid indole alkaloids consist of an indole ring and a secologanin. Among the key enzymes in the metabolic pathway of this class is the enzyme strictosidine synthase, and its product, strictosidine, is an important branching point in this pathway. It can be further transformed into various related alkaloids like ajmalicine, quinine, and strychnine [15]. In the biosynthesis pathway of tetrahydrophenylisoquinoline alkaloids, the branching point is (S)-reticuline. Under the action of specific synthases, it can be further converted into alkaloids such as berberine, sanguinarine, and morphine [16]. Precursors for alkaloids like nicotine and scopolamine are derived from ornithine or arginine. Key enzymes involved in the biosynthesis of these compounds include ornithine decarboxylase, tropinone reductase, and hyoscyamine hydroxylase [17]. Since the isolation of the first alkaloid, morphine, from opium in 1816, a variety of alkaloids such as piperine, atropine, caffeine, and quinine have been sequentially isolated. Currently, there are over 27,000 reported alkaloids with diverse chemical structures. Among these, 88 of them have various pharmacological properties, including analgesic (morphine), stimulant (caffeine and ephedrine), psychoactive (mescaline and cocaine), antimicrobial (quinine), and anticancer (vinblastine and vincristine) activities [18].

The plant kingdom comprises more than 8000 to 10,000 phenolic compounds, which exhibit excellent antioxidant activity due to one or more aromatic rings with hydroxyl groups. Additionally, they serve as defense compounds in plants against bacteria, fungi, and insects [19]. Phenolic compounds can be further divided into four classes: flavonoids, phenolic acids that derived from hydroxybenzoic acids or hydroxycinnamic acids, stilbenes, and lignans [20]. Flavonoids originate from the phenylpropanoid metabolic pathway, with their basic structure composed of a C6-C3-C6 unit formed by connecting two phenolic ring moieties (A and B rings) with three carbon atoms. The C3 part can be a linear chain or form a five-membered or six-membered oxygen heterocycle with the C6 portion. Depending on differences in whether the C3 portion is cyclized, the degree of oxidation, and the substitution patterns, flavonoids can be further classified into various subclasses, including chalcones, flavones, anthocyanidins, flavanones, flavonols, and isoflavones [21]. Phenolic compounds in plants have various biosynthetic pathways, with the biosynthesis of flavonoids being one of the more extensively studied and understood processes. First, phenylalanine ammonia lyase, as the first crucial enzyme in plant phenolic compound production, catalyzes the deamination of phenylalanine to form trans-cinnamic acid. Subsequently, under the action of cinnamate 4-hydroxylase, cinnamic acid is hydroxylated, forming p-coumaric acid. Then, p-coumaric acid, in conjunction with coenzyme A, is converted to p-coumaroyl-CoA by the action of 4-coumaroyl:CoA ligase. This p-coumaroyl-CoA is further catalyzed to form chalcone when combined with malonyl-CoA, and this product serves as a precursor for various flavonoid compounds (Figure 1C). Many natural phenolic compounds are beneficial for human health. Some extensively researched examples include resveratrol, a stilbene found in grapes and red wine; epigallocatechin-3-gallate, a flavanol found in tea; and quercetin and rutin, flavonols present in various plants such as tea, onions, and Ginkgo biloba [21].

## 3. Production of Plant Secondary Metabolites Using Natural Hosts

Heterologous synthesis in hosts like *N. benthamiana*, tomatoes, and rice is primarily aimed at elucidating and confirming the synthetic pathways or enhancing crop nutrition, and the yields are generally low [6]. Natural hosts for secondary metabolites inherently possess complete metabolic pathways, and their cells may be more tolerant of the accumulation of their own secondary metabolites due to the presence of compartmentalization, metabolic enzymes, and transport systems. These allow for direct in vitro cultivation to produce secondary metabolites.

### 3.1. Comparison of Different In Vitro Plant Culture Systems

Currently, many different types of plant secondary metabolites have been successfully produced through in vitro cultivation, including suspension cells, adventitious roots, and hairy roots (Table 1). In comparison to traditional cultivation and natural plant growth, in vitro plant culture systems offer several advantages: not influenced by geographical or seasonal changes; provides a controlled system, ensuring the continuity, uniformity, and yield of products; allows for the isolation and purification of target compounds from the culture medium, reducing downstream costs; permits control over cell growth and regulation of metabolic processes.

Suspension cells, similar to microorganisms, grow as single cells or small cell clusters. Therefore, when it comes to scaling up production, they can directly inherit the experience and equipment used in microbial fermentation, making them easier to work with compared to tissue and organ cultures. Historically, several alkaloids, such as berberine (13.2% dry weight), polyphenols like shikonin (12% dry weight), and diterpenes like Taxol (75,000 L), have been successfully produced on an industrial scale using suspension cell cultures [22,23,24,25]. In addition, triterpenoid ginsenosides, as well as other polyphenolic compounds like resveratrol, shikonin, and rosmarinic acid, can also be efficiently synthesized in suspension cells, achieving production levels of up to grams per liter (Table 1).

**Table 1:** Typical examples of plant secondary metabolites production in plant chassis.

Compound	Product	Host	Strategy or Observations	Platform	Titer	Vessel	Ref.
Terpenoids							
Monoterpenes	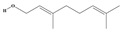 Geraniol	*Nicotiana tabacum*	Stable transgene of *Valeriana officinalis* geraniol synthase; Culture condition optimization.	Suspension cells	5.2 mg/L	Flask	[26]
		*Nicotiana tabacum*	Stable transgene of *Valeriana officinalis* geraniol synthase	Hairy roots	0.11 mg/L	Wave-mixed bag bioreactor	[27]
	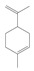 Limonene	*Mentha piperita*	Overexpression of limonene synthase and co-suppression of limonene-3-hydroxylase	Leaves	3.87 mg/g FW (79% of total essential oil)	Flask	[28]
		*Nicotiana tabacum*	Overexpression of plastid targeting amorpha-4,11-diene synthase and FPP synthase	Leaves	0.5 μg/g FW	Flask	[29]
		*Calotropis procera*	Fe_3_O_4_ nanoparticles and salicylic acid reduced limonene contents	Hairy roots	7.9% of total essential oil	Flask	[30]
	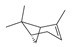 α-pinene	*Levisticum officinale*	Optimization of culture medium	Hairy roots	1.3% of total essential oil	Flask	[31]
	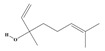 Linalool	*Bursera linanoe*	Screened 6-benzylaminopurine concentration for callus induction	Suspension cells	3.02 mg/g DW	Stirred tank bioreactor	[32]
Sesquiterpenes	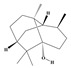 Patchoulol	*Pogostemon cablin*	Transient overexpression of a MYB transcription factor	Leaves	2.8 mg/g FW	-	[33]
		*Physcomitrella patens*	Overexpression of patchoulol synthase and HMGR gene	Moss tissues	1.34 mg/g DW	-	[34]
	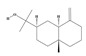 β-eudesmol	*Atractylodes lancea*	Elicitation with extracts from endophytic fungi	Suspension cells	63 μg/g FW	Flask	[35]
		*Atractylodes lancea*	-	Hairy roots	5 μg/g FW	Flask	[36]
	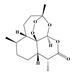 Artemisinin	*Artemisia annua*	Elicitation by oligosaccharide from *Fusarium oxysporum* mycelium combined with NO donor sodium nitroprusside	Hairy roots	28.5 mg/L	Flask	[37]
		*Artemisia annua*	Design of bioreactor	Shoot culture	48.2 mg/L	Nutrient mist bioreactor	[38]
		*Artemisia annua*	Coronatine pretreatment and addition of 30 g/L sorbitol	Suspension cells	9.33 mg/L	Flask	[39]
Essential oil		*Anethum graveolens*	-	Hairy roots	0.02% *v*/*w* FW	Flask	[40]
		*Achillea millefolium*	Addition of Miglyol 812	Suspension cells	0.002%, *w*/*w* FW	Flask	[41]
		*Achillea millefolium*	-	Hairy roots	0.05% *v*/*w* FW	Flask	[42]
		*Levisticum officinale*	Optimization of culture medium	Hairy roots	0.018 *v*/*w* FW	Flask	[31]
Diterpenes	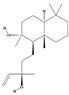 Sclareol	*Physcomitrella patens*	Heterologous overexpression of two sclareol synthase genes	Moss tissues	2.28 mg/L	-	[43]
	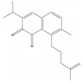 Aethiopinone	*Salvia sclarea*	Overexpression of CPPS	Hairy roots	208.98 mg/L	Flask	[44]
	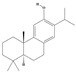 Ferruginol				230.4 mg/L		
	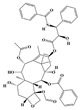 Paclitaxel	*Taxus chinensis*	Elicitation and optimization of culture conditions	Suspension cells	902 mg/L	Stirred tank bioreactor	[45]
		*Taxus Media*	Elicitation by MeJA, sodium nitroprusside, and L-phenylalanine	Hairy roots	3.18 mg/g DW	Flask	[46]
	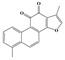 Tanshinone	*Salvia miltiorrhiza*	Overexpression of *SmGGPPS* and *SmDXSII*	Hairy roots	12.93 mg/g DW	Flask	[47]
Triterpenes	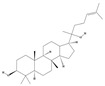 Ginsenosides	*Panax ginseng*	Overexpression of *PgFPS*	Hairy roots	36.42 mg/g DW	Flask	[48]
		*Panax ginseng*	Elicitation by nitrogen-fixing bacteria	Adventitious roots	105.58 mg/g DW	Flask	[49]
		*Panax sikkimensis*	Elicitation with culture filtrates from bacteria	Suspension cells	222.2 mg/L	Flask	[50]
		*Panax notoginseng*	Addition of conditioned medium	Suspension cells	3.12 g/L	Air-lift bioreactor	[51]
	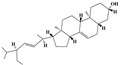 α-spinasterol	*Platycodon grandifolium*	Overexpression of *PgHMGR*	Hairy roots	1.78 mg/g DW	Flask	[52]
	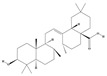 Oleanolic acid	*Calendula officinalis*	Elicitation by jasmonic acid	Hairy roots	52.52 mg/g DW	Flask	[53]
		*Calendula officinalis*	Elicitation by jasmonic acid	Suspension cells	0.84 mg/g DW	Flask	[54]
	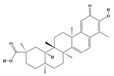 Celastrol	*Tripterygium wilfordii*	Overexpression of *TwSQS2*	Hairy roots	2.41 mg/g DW	Flask	[55]
Tetraterpenes	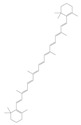 β-carotene	*Morus indica*	Overexpression of β-carotene hydroxylase 1 and treated with high light	Plants	256 mg/g FW	Flask	[56]
		*Daucus carota*	Optimization of culture medium	Suspension cells	2.19 mg/g DW	Flask	[57]
	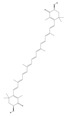 Astaxanthin	*Solanum lycopersicum*	Co-expression of the algal β-carotene ketolase from *Chlamydomonas reinhardtii* and β-carotene hydroxylase from *Haematococcus pluvialis*	Plant fruits	16.1 mg/g DW		[58]
Phenols							
Anthocyanins	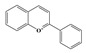 Anthocyanin	*Perilla frutescens*	Optimization of sugar supply and culture modes	Suspension cells	5.8 g/L	Flask	[59]
		*Nicotiana tabacum*	Co-expression of the MYB and bHLH transcription factors from *Antirrhinum majus*	Suspension cells	25 mg/g DW	Flask and small stirred tank	[60]
		*Aralia cordata*	Administration of CO_2_	Suspension cells	1.09 g/L	Jar fermentor	[61]
Proanthocyanidin	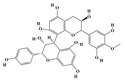 Proanthocyanidin	*Litchi chinensis*	Overexpression of *LcMYB1*	Hairy roots	15 mg/g FW	Flask	[62]
	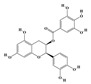 (-)-Epicatechin 3-O-gallate	*Fagopyrum esculentum*	-	Hairy roots	10 mg/g DW	Flask	[63]
	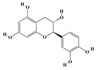 (+)-catechin	*Taxus cuspidata*	Elicitation with MeJA	Suspension cells	34 mg/g DW	Flask	[64]
	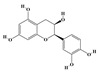 (-)-epicatechin				52 mg/g DW		
Flavanols	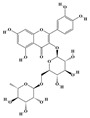 Rutin	*Fagopyrum tataricum*	Overexpression of *UGT73BE5*	Hairy roots	80 mg/g DW	Flask	[65]
	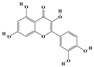 Quercetin	*Polygonum multiflorum*	Elicitation by MeJA	Hairy roots	17.58 mg/g DW	Flask	[66]
		*Polygonum multiflorum*	Elicitation by jasmonic acid	Suspension cells	0.16 mg/g DW	Flask	[67]
		*Polygonum multiflorum*	Optimization of culture parameters	Adventitious roots	3.5 mg/g DW	Balloon-type bubble (air-lift) bioreactor	[68]
	Rutin	*Ficus deltoidea*	Medium optimization	Suspension cells	39.13 mg/g DW	Flask	[69]
	Quercetin				3.92 mg/g DW		
Isoflavones	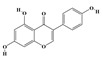 Genistein	*Trifolium pratense*	Optimization of light conditions	Hairy roots	2.45 mg/g DW	Flask	[70]
		*Psoralea corylifolia*	Elicitation with spermidine	Suspension cells	4.75 mg/g DW	Flask	[71]
Stilbenoids	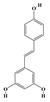 Resveratrol	*Arachis hypogaea* L.	Elicited with MeJA and cyclodextrin	Hairy roots	5.3 mg/g DW	Flask	[72]
		*Vitis vinifera*	Elicitation by MeJA	Suspension cells	209 mg/L	Stirred bioreactor	[73]
		*Vitis vinifera*	Treated with modified β-cyclodextrin	Suspension cells	5 g/L	Flask	[74]
Naphthoquinone	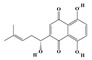 Shikonin	*Lithospermum erythrorhizon*	Optimization of culture parameters	Suspension cells	4 g/L	Stirred tank bioreactor	[24]
Hydroxycinnamic acids	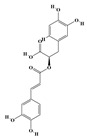 Rosmarinic acid	*Anchusa officinalis*	High-density culture with optimized culture parameters	Suspension cells	3.7 g/L	Stirred tank bioreactor	[75]
	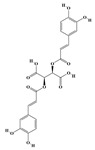 Chicoric acid	*Echinacea purpurea*	Addition of 2 mg/L indole butyric acid	Adventitious roots	22 mg/g DW	Air-lift bioreactor	[76]
	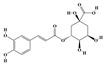 Chlorogenic acid				5 mg/g DW		
Alkaloids							
Tetrahydroisoquinoline alkaloids	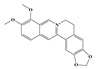 Berberine	*Thalictrum minus*	Cells immobilized in calcium alginate beads	Suspension cells	875 mg/L	Modified bioreactor	[77]
		*Coptis japonica*	Optimization of culture parameters	Suspension cells	3.5 g/L	Stirred bioreactor	[23]
Tropane alkaloids	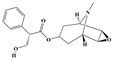 Scopolamine	*Duboisia myoporoides*	Repeated selection of transformed root lines	Hairy roots	32 mg/g DW	Flask	[78]
Pyridine alkaloids	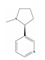 Nicotine	*Nicotiana tabacum*	Optimization of culture parameters	Hairy roots	83.3 mg/L	Stirred bioreactor	[79]
		*Nicotiana tabacum*	Optimization of culture parameters	Suspension cells	780 μg/g FW	Flask	[80]
Quinoline alkaloids	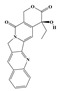 Camptothecin	*Ophiorrhiza pumila*	In situ absorption by polystyrene resin	Hairy roots	3.2 mg/L	Flask	[81]
		*Ophiorrhiza pumila*	Overexpression of *OpG10H* and *OpSLS*	Hairy roots	3.5mg/g FW	Flask	[82]
BenzylIsoquinoline alkaloids	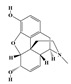 Morphine	*Papaver somniferum*	Removal of hormones from the medium	Suspension cells	2.5 mg/g DW	Flask	[83]
		*Papaver somniferum*	-	Hairy roots	2.6 mg/g DW	Flask	[84]
		*Papaver bracteatum*	Elicitation by hormone indole-3-acetic acid	Suspension cells	243.2 mg/g FW	Flask	[85]
		*Papaver bracteatum*	Overexpression of codeinone reductase	Hairy roots	2.8 mg/g DW	Flask	[86]
		*Papaver orientale*	Elicitation with salicylic acid	Hairy roots	2.9 mg/g DW	Flask	[87]
Terpenoid indole alkaloid	Total terpenoid indole alkaloids 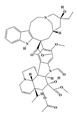 Vincristine	*Catharanthus roseus*	Overexpression of *ORCA3* with precursors feeding	Hairy roots	5 mg/g DW	Flask	[88]
		*Catharanthus roseus*	Co-overexpression of *ORCA3* and *SGD* induced with 3 μM dexamethasone	Hairy roots	10 mg/g DW	Flask	[89]

The synthesis of plant secondary metabolites is often subject to complex spatial (subcellular structures, tissues, organs, and entire plants) and temporal (different developmental stages) regulation. From an evolutionary perspective, this synthesis and regulation in plants are significant because they help avoid self-intoxication, enable better control of metabolic flux (by adding additional regulation and reducing feedback inhibition), and provide enzymes with more suitable catalytic environments. This complexity makes the synthesis of metabolites with complex biosynthetic pathways within a single cell a significant challenge. Selecting a high-yielding and fast-growing suspension cell line is a crucial step. Taking Taxol production as an example, among the eight species of *Taxus* and two hybrids, *T. baccata* and *T. chinensis* are the main high-yield producers [45,90]. Similarly, for morphine production, the suspension cell line from *Papaver bracteatum* can achieve an approximately 10 times higher synthesis of morphine compared to *P. somniferum*, 243.2 mg/g FW and 2.5 mg/g DW, respectively [83,85]. Aside from the influence of plant species, as suspension cell lines are sub-cultured, they often undergo varying degrees of differentiation, which can also affect yield stability. Additionally, the reduction in yield after long-term cultivation is also related to epigenetics [91]. In a study on the production of indole alkaloids by *Catharanthus roseus* suspension cells, it was found that, after 8 years of continuous cultivation, the total yield gradually decreased. However, researchers were still able to identify some cells with highly productive phenotypes, demonstrating that the initial callus tissue is the source of subsequent heterogeneity [92]. Lee et al. [93] partially addressed this issue by isolating and culturing undifferentiated cambial meristematic cells from *T. cuspidata*, achieving a high content of 263 mg/kg FW. Nevertheless, plant stem cell isolation technology, particularly for herbaceous plants, still requires improvement, and therefore, this technology has not seen more widespread application.

Plant organs have specialized tissue structures and do not experience the yield instability caused by the redifferentiation of suspension cells [94]. Particularly, roots have been found in recent years to be capable of achieving higher yields compared to their corresponding suspension cell lines. Industrial productions of ginsenosides (20,000 L) using adventitious roots of *Panax ginseng* has already been achieved [95]. Moreover, various compounds such as artemisinin in *Artemisia annua*, oleanolic acid in *Calendula officinalis*, quercetin in *Polygonum multiflorum*, genistein in *Trifolium pratense*, oleanolic acid in *Calendula officinalis*, celastrol in *Tripterygium wilfordii*, tropane alkaloids in *Duboisia* [96], pyridine alkaloids in *Nicotiana tabacum*, as well as terpenoid indole alkaloids and quinoline alkaloids, all show higher yields in hairy roots (Table 1).

However, roots are not beneficial for the production of all secondary metabolites. Some secondary metabolites show no significant difference in content between hairy and natural roots, such as tanshinone in *Salvia miltiorrhiza* [97], and benzyl isoquinoline alkaloids in *P. somniferum* (opium poppy) and *Eschscholzia californica* [98]. Compared to suspension cells, the content of the sesquiterpene β-eudesmol in *Atractylodes lancea* adventitious roots is lower than that in suspension cells (5 and 18 μg/g FW, respectively) [35,36]. The content of benzyl isoquinoline alkaloids, such as morphine, in *P. bracteatum* is also higher in suspension cells compared to hairy roots. Berberine, shikonin, and Taxol, as mentioned earlier, also have significantly higher content in suspension cells compared to roots. Adventitious roots and hairy roots exhibit comparable abilities in the synthesis of many secondary metabolites, but the latter often demonstrate a more stable morphology, possibly due to the integration of plant hormone biosynthetic genes from *Agrobacterium rhizogenes* into the genomes. For instance, in our study, the adventitious roots of *Tripterygium wilfordii* tend to aggregate and undergo partial dedifferentiation [99], while hairy roots do not [100]. Additionally, unlike adventitious roots, hairy roots appear to have lower capability for secreting secondary metabolites into the culture medium, such as the indole alkaloids of *Catharanthus roseus* [101], tropane alkaloids of *Duboisia leichhardtii* [102], sesquiterpene pyridine alkaloids of *Tripterygium wilfordii* [103], terpenoids of *Salvia miltiorrhiza* [104], and red beet pigments [105]. This difference may also be attributed to the genetic manipulation of hairy roots.

Comparing suspension cells and root cultures, it appears that structurally complex compounds often require cell organization with differentiation, as found in root or organ forms, to be effectively synthesized. This is particularly true for various classes of alkaloids and triterpenes. However, some structurally simple secondary metabolites, like essential oils, do not consistently demonstrate high production in suspension cells [31,40]. Due to the diverse sources of suspension cells and the significant variation in metabolite production across different tissues, it is not easy to establish a universal principle based on existing data. Therefore, for specific secondary metabolites, screening in different in vitro culture systems remains necessary.

### 3.2. Enhancing the Yield of Plant in Vitro Culture Systems by Process Optimization

Due to the close connection between primary and secondary metabolism in plants, any factor that affects primary metabolism may also influence secondary metabolism. Therefore, adjusting the growth environment can further enhance the yield of secondary metabolites. This can include optimizing the types and concentrations of nutrients and plant hormones in the culture medium, as well as optimizing aeration rate, pH, and inoculum size [4]. Additionally, the addition of precursors, elicitors, and immobilization techniques may also further increase production [106]. In particular, methods that mimic biotic stress have been widely applied [107,108]. For example, jasmonic acid and its methyl derivative, methyl jasmonate (MeJA), which are important plant stress signaling molecules, can induce the biosynthesis of defensive compounds [109]. Secondary metabolites secreted into the culture medium may be degraded by metabolic enzymes in the medium, and the accumulated secondary metabolites themselves could be harmful to plant cells and/or to the feedback inhibition of biosynthesis. Therefore, various methods, such as in situ adsorption and two-phase culture, should be employed to remove the products from the culture medium promptly [106,110,111].

The successful commercial production of plant-based secondary metabolites depends on the scaling up of the process from shake flasks to bioreactors. Stirred-tank reactors and airlift reactors are widely used in cell suspension cultures [112]. The former are known for their simple scalability, strong oxygen supply, and uniform mixing, but they have the disadvantage of high shear forces [113]. The latter are easy to scale up, have low operating costs and contamination rates, and generate low shear forces. However, they suffer from poor oxygen mass transfer, non-uniform mixing, and a propensity for foaming [114]. The cultivation of root tissues primarily involves the use of mist bioreactors [115] and balloon-type bubble bioreactors [116]. The research group led by Kee-Yoeup Paek in South Korea successfully used a balloon-type bubble bioreactor to scale up the cultivation of *Panax ginseng* hairy roots to volumes ranging from 10,000 to 20,000 L [117]. Temporary immersion bioreactors and root zone immersion bioreactors are suitable for explant culture. Rotating drum bioreactors can be used for the culture of suspension cells, root tissues, and explants, but they face challenges when scaling up, and their heat transfer performance is low. Other reactor types, such as disposable reactors, membrane bioreactors, and wave bioreactors, are challenging to scale up due to their specific characteristics and are primarily suitable for small-scale cultivation [112,118]. More information on the structure and design of plant bioreactors can be found in the article by Murthy et al. [119].

### 3.3. Plant Metabolic Engineering

The complexity of plant secondary metabolite biosynthetic pathways results in a diverse range of strategies for manipulating metabolic pathways [120], including utilizing gene editing tools, such as Clustered, Regularly Interspaced, Short Palindromic Repeats (CRISPR)/dead CRISPR associated protein 9 (dCAS9), to activate single or multiple-target genes; reducing the expression of genes in competitive pathways through techniques such as RNA interference (RNAi) and CRISPR/CAS9; modifying the expression of transcription factors that control multiple biosynthetic genes; and enhancing the compartmentalization and/or release of the desired product by transporter engineering.

Enhancing the activity of key enzymes controlling the biosynthesis of a specific secondary metabolite through genetic engineering can increase the content of the target secondary metabolite in transgenic plants. For example, overexpressing FPP synthase, squalene synthase, or the upstream 3-hydroxy-3-methylglutaryl-coenzyme A reductase (HMGR) in the mevalonate pathway (MVA), for example, can increase the production of downstream terpenoid end-products. However, due to the presence of downstream branch pathways, increasing the expression of upstream pathway enzyme genes alone may not achieve the accumulation of the target compound. For instance, overexpressing the first two genes, tabersonine 16-hydroxylase (T16H) and 16-O-methyl transferase (16OMT), in *C. roseus* hairy roots failed to produce the target vindoline [121]. Using RNAi to disrupt undesired metabolic branches can redirect synthetic metabolism toward the target. Allen et al. [122] used this technique to silence the gene family of codeinone reductase (COR) in poppies, blocking the synthesis of opioid substances while leading to the significant accumulation of the precursor to codeine, (S)-reticuline. Overexpressing limonene synthase while inhibiting the expression of by-product synthetic gene limonene-3-hydroxylase substantially increased the content of limonene and its proportion in the total essential oil (up to 79%) [28].

The activity and expression of these biosynthetic enzymes are regulated by corresponding transcription factors and other regulatory genes, making metabolic engineering of transcription factors potentially highly effective [123]. Currently, transcription factors involved in plant secondary metabolite biosynthetic pathways mainly include WRKY, myeloblastosis (MYB), basic helix–loop–helix (bHLH), APETALA2/Ethylene-Responsive-Factor (AP2/ERF), jasmonate-responsive ERF (JRE), basic Leucine Zipper (bZIP), SQUAMOSA promoter-binding protein-like (SPL), and YABBY classes, among others [11,124]. Taking the indole alkaloid biosynthetic pathway as an example, the overexpression of ORCA3 together with the addition of the terpene precursor [88] or overexpression of downstream key enzyme genes [89] can increase the final yield of the target compound.

The excessive accumulation of secondary metabolites within cells can have two main effects: on one hand, it can increase cellular toxicity and hinder cell growth; on the other hand, it can result in the feedback inhibition of synthetic enzyme activity, reducing overall biosynthesis [125,126]. Metabolic flux balance analysis indicates that the extracellular secretion and transfer of secondary metabolites are among the key limiting steps for improving yield [127]. Additionally, due to the simplicity of the culture medium, the economic aspects of purifying secondary metabolites through medium separation are favorable. Avoiding cell disruption can further save on cultivation and production costs [128]. Therefore, enhancing the extracellular secretion of secondary metabolites through genetic engineering is of significant importance in plant or microbial biosynthesis research [127,129]. Two main types of proteins have been identified to be involved in the transport of plant secondary metabolites: ABC (ATP-binding cassette) and MATE (Multidrug and toxic compound extrusion) [130,131]. Among these, ABC transport proteins exhibit higher substrate specificity compared to MATE, and they are more frequently associated with plant disease resistance [132], especially the pleiotropic drug resistance (PDR) transport proteins [133,134]. Pomahačová et al. [135] heterologously expressed the transporter gene CjMDR1, which mediates the intracellular transport of berberine, in *C. roseus*. This led to an increase in the intracellular content of ajmalicine and tetrahydroalstonine in suspension cell lines. Our research group successfully enhanced the extracellular secretion of sesquiterpene pyridine alkaloids and triptolide in *Tripterygium wilfordii* by regulating influx and efflux transporter proteins, respectively [103,136]. However, the application of transporter proteins in metabolic engineering is still limited, as only a small number of plant secondary metabolite transporter proteins have been functionally characterized, and research on the post-translational regulation of transporter protein activity is lacking [137]. Our recent study elucidated the phosphorylation regulation mechanism of the AtPDR6 transporter protein, which mediates the extracellular secretion of camalexin in *Arabidopsis thaliana* [138], laying a foundation for subsequent transporter engineering.

### 3.4. Multigene Transformation of Plants

Plant secondary metabolism involves the expression of multiple genes, and enhancing the co-expression of multiple genes is essential for further increasing the yield of secondary metabolites. The invention of large DNA fragment assembly technologies has made the construction of large fragment vectors more convenient. Some widely used techniques include Zinc Finger Nuclease and Homing Endonuclease-Mediated Assembly [139], ligation cycling reaction [140], Golden Gate cloning [141], Cre/loxP recombination [142], MultiRound Gateway recombination [143], Gibson Assembly [144], and yeast assembly [145]. However, due to the possibility of multi-gene silencing, rearrangement, or internal interactions in plants [146], successfully transforming vectors containing hundreds of thousands of base pairs into plant cells and achieving stable expression in plants, especially non-model plants, is one of the major challenges in plant metabolic engineering [147,148]. The mainstream methods of introducing large DNA into plants include particle bombardment, polyethylene glycol-mediated protoplast transformation, and *Agrobacterium*-mediated transformation. The first two methods involve the induction of callus tissue or regeneration of the cell wall, which can be challenging for many plants. For the last method, *Agrobacterium* cannot infect many medicinal plants or has very low efficiency [149]. Additionally, when co-expressing multiple genes, it is important to adjust the strength of gene promoters and carefully choose the insertion sites to prevent gene silencing [150]. Placing multiple pathway enzyme genes into artificial plant chromosomes can help avoid position effects associated with genome insertion, potentially achieving stable expression [151,152]. However, this technology still faces challenges, such as the potential inability to form active centromeres and the risk of abnormal plant growth or death [153].

### 3.5. Elucidation of Biosynthetic Pathway

All of the aforementioned strategies for metabolic regulation require an in-depth understanding of the biosynthetic pathways of secondary metabolites. The redundancy of pathway enzyme genes and challenges in substrate accumulation or chemical synthesis hinder the specific identification of enzyme genes for certain steps [8,154], and only widely studied natural products have been fully elucidated (Figure 1). Comparing gene expression levels between different biotic/abiotic stresses, elicitors, and tissues is an effective strategy for identifying biosynthetic genes. These co-expression data are typically analyzed through linear correlation analyses, such as linear regression and/or hierarchical clustering based on Euclidean distance, to extract potential genes with similar functions to the “bait” gene [155].

In recent years, the functional elucidation of candidate genes in a biosynthetic pathway can be achieved by expressing them constitutively in *N. benthamiana*, without the need to consider synthesis order or prepare large amounts of intermediates. For example, Caputi et al. [156] identified the enzymes responsible for synthesizing tabersonine 1 and catharanthine 3 by overexpressing three enzymes, precondylocarpine acetate synthase, dihydroprecondylocarpine synthase, and tabersonine synthase/catharanthine synthase, in *N. benthamiana*. By rerouting diterpene biosynthesis from the chloroplast to the cytosol, De La Peña and Sattely [157] achieved the complete elucidation of the momilactone biosynthetic pathway in tobacco. However, due to the influence of endogenous metabolic pathways on the “orthogonality” of the transgenic pathway in *N. benthamiana* and its lack of accumulation of some necessary precursors, it often requires the simultaneous infiltration of precursor molecules during transient transformation or the use of other hosts. Metabolic engineering operations in yeast can help construct synthesis pathways for pathway intermediates or test the function of specific enzymes by adding substrates. For example, using this approach, the P450 enzyme CYP71AV1 involved in the biosynthesis of artemisinin was functionally identified, and the precursor, artemisinic acid, was abundantly produced [11].

## 4. Production of Plant Secondary Metabolites Using Microbial Chassis

Compared to plant hosts, microorganisms have gained popularity in recent years due to their faster growth rates, simpler and clearer metabolic networks, ease of genetic transformation, and scalability (Table 2). Among microorganisms, *Escherichia coli* and yeast are more widely used than others, mainly due to their well-defined metabolic pathways, more stable gene expression systems, and efficient, abundant biosynthetic elements [7].

### 4.1. Engineering Enzymes and Cofactors

Once the biosynthetic pathway of plant secondary metabolites is elucidated, the genes encoding pathway enzymes can theoretically be introduced into microorganisms to produce these secondary metabolites. However, enzymes from the native host may have no activity or poor activity in microorganisms. Through genome and transcriptome mining, homologous enzymes or isoenzymes with potential catalytic activity can be identified and screened. For example, Cheng et al. [162] identified the most active limonene synthase from nine candidate enzymes obtained from the genera *Citrus*, *Solanum habrochaites*, and *Agastache rugosa*. Jiang et al. [159] discovered an enzyme with higher activity from *C. roseus* through the screening of nine geraniol synthase genes from diverse species. Additionally, there is a wealth of metagenomic resources in unculturable environmental microorganisms that can be explored for genes encoding enzymes with potential activities [208].

After obtaining candidate pathway enzyme genes, combinatorial optimization can be used to find the optimal combination. For example, Clomburg et al. [209] combined genes from *Staphylococcus aureus*, *Myxococcus xanthus*, *Clostridium beijerinckii*, and *E. coli* to construct an isoprenoid alcohol pathway using a retrobiosynthetic approach to provide a high accumulation of precursors for downstream terpene synthesis. By expressing four biosynthetic enzymes from three different plants, Kim [187] achieved 18.6 mg/L of genistein from p-coumaric acid in *E. coli*. For synthesizing lycopene, Shi et al. [181] initially screened the activities of nine GGPP synthase, five CrtB, and seven CrtI enzymes from various hosts, followed by further screening of over thirty combinations. This process led to a significant increase in lycopene production, from 4.5 mg/L to 118 mg/L. However, this combinatorial optimization approach often involves a considerable amount of trial-and-error efforts. Fortunately, gene mining aided by bioinformatics [210] and the convenience of component assembly in synthetic biology [211] can reduce the initial screening and vector construction work.

If the enzymes discovered in microorganisms exhibit issues such as insufficient substrate specificity, known as substrate promiscuity, low activity, and poor stability, they can be modified using enzyme engineering techniques such as random mutagenesis, directed evolution, and computer-aided semi-rational design, to alter active pockets, replace amino acids in loop regions, or make other modifications. In yeast, ERG20 catalyzes the condensation of IPP and DMAPP into both GPP and FPP. Ignea et al. [212,213] introduced mutations into ERG20 using rational design, changing it into sole GPP synthase or GGPP synthase. Sometimes, the activity and (or) stability of enzymes can also be improved by fusing them with tag proteins or other enzymes. For instance, terpene synthase and lavandulyl diphosphate synthase fused with maltose-binding protein exhibited improved stability [169]. In their study, it was also found that the fused enzymes showed higher activity compared to individual expression, which was mainly due to the closely located enzymes which allowed for a more efficient substrate transfer. Zhang et al. [172] found that GGPPS and taxadiene synthase exhibit different subcellular localization in yeast and that fusion expression increases taxadiene production by 54%.

When expressing plant-derived enzymes in microorganisms, especially P450 enzymes, it is common to encounter reduced or lost activity. Approximately 40% of plant-derived P450 enzymes are expressed poorly in yeast [214]. Additionally, P450 enzymes typically require characteristic redox partners, such as cytochrome P450 reductase (CPR), to transfer electrons. However, microorganisms may lack a sufficient amount of CPR or the available CPR may not effectively couple with exogenous P450 enzymes. Possible strategies to address this issue include adjusting the expression level of P450 enzyme genes and engineering them [176], screening for better CPRs [177], and taking inspiration from the natural P450/CPR fusion observed in BM3 (CYP102A1) from *Bacillus megaterium* by fusing P450 enzymes with CPR [171,187].

As essential compounds for the activity of various exogenous enzymes, cofactors often face competition with endogenous enzymes, which often leads to inadequate cofactor supply and subsequent growth retardant. Due to the involvement of approximately 1600 enzyme-catalyzed reactions, the NAD(P)H/NAD(P)+ and ATP/ADP systems have attracted widespread attention [215]. Adjusting the expression of three genes in the central metabolic module of *E. coli*: α-ketoglutarate dehydrogenase, succinate dehydrogenase, and transaldolase B enhanced carbon flux toward the TCA cycle and the pentose phosphate module, thereby increasing the supply of NADPH and ATP [182]. Similarly, in yeast, NADPH supply can be increased by reducing the glycolytic pathway and enhancing the pentose phosphate pathway [216]. Using this strategy, Cao et al. [169] significantly increased the synthesis of the diterpene sclareol. Also in yeast, the NADH kinase POS5 can convert NADH to NADPH, and the overexpression of this gene can increase NADPH supply [181]. In addition to supplementing cofactors, it is also possible to block endogenous cofactor synthesis pathways in the host and then use the modified host for high-throughput screening of target enzymes. This approach may yield enzyme variants that allow for cofactor regeneration without affecting growth [217]. It is noteworthy that cofactors are typically synthesized and stored in specific organelles in the native host, which further complicates the challenge of expressing active P450 enzymes in microorganisms. For example, heme, primarily found in mitochondria, can hinder the construction of P450 enzyme synthesis pathways in the cytoplasm [218].

### 4.2. Pathway Establishment and Optimization

Microbial metabolic networks have developed tight regulation to maintain metabolic homeostasis, and introducing exogenous synthetic pathways may lead to disruptions in endogenous metabolism, increase cellular metabolic load, and the introduction of new substances that are toxic to cells. Therefore, it is essential to carefully design synthetic pathways to balance the overall metabolic flux and cofactor levels [219].

Before constructing a pathway, it is important to consider the form in which exogenous DNA exists in microorganisms. While integrating exogenous DNA into the genome is ideal due to the instability of plasmids and variations in copy number, many laboratory-scale studies still use plasmids due to their convenience. Traditional homologous recombination systems using counter-selection markers are time-consuming, so site-specific recombination and transposon-mediated random insertion are often used. The former is widely used in yeast, utilizing sites such as nutrient and amino acid synthesis gene loci, delta sites, and rDNA sites. The CRISPR/Cas system can induce site-specific breaks, allowing for knockouts, in-del mutations, and the insertion of exogenous DNA. Coupling specific sites with CRISPR/Cas9 can enable a higher insertion copy number, potentially increasing production further [220].

Manipulating individual or combinations of genes remains labor-intensive and iterative. Each change that leads to a change in phenotype needs to be considered in the next step. The ideal approach involves multiplex genome engineering, such as oligo-based, recombinase-based, and CRISPR-based methods, along with biosensors and high-throughput screening tools [221]. However, this also increases uncertainty and operational complexity, so it is not widely applied in the successful cases we have investigated.

Similar to plant metabolic engineering, microbial strategies mainly include the regulation of pathway enzyme gene expression, inhibition of branches, and control of transport. The expression levels of pathway enzyme genes are one of the crucial factors influencing product synthesis. Many studies choose to overexpress the enzyme genes responsible for precursor supply pathways to increase the capacity of precursor pools. For example, to increase upstream products of terpenoids, IDI1 and HMGR are often overexpressed [159,160]. However, it is worth noting that, during the early stages of cultivation, the overaccumulation of precursors or end products may have adverse effects on cell growth, such as precursors of terpenoids and polyphenols [160,222]. At the same time, the early expression of exogenous pathways can slow down cell growth due to increased metabolic burden. In such cases, the dynamic control of gene expression can be chosen. In the study of Xie et al. [222], the expression of genes involved in the MVA pathway and squalene synthesis pathway initially remained at normal levels. It was only after the cells reached high density that these genes were overexpressed, achieving a form of sequential control. Dynamic control is often achieved through condition- or inducible-type promoters that regulate gene expression, such as GAL promoters [223] or hexose transporter promoters [162].

Dealing with nonlinear diverging–converging metabolic pathways often involves a more precise control of the supply of two or more precursors, and adjusting the metabolic flux in one branch may affect the others. This requires laborious work to optimize gene copy numbers, expression levels, ribosome binding sites, and other factors. Separating diverging pathways and building them separately in different strains can reduce metabolic stress, create distinct cellular environments, and minimize interference from unwanted pathway modules. For example, the synthesis of rosmarinic acid was divided into three *E. coli* strains to provide two precursors and downstream pathways. By optimizing the balance of these three strains and the composition of the culture medium, the rosmarinic acid content was increased from 4.5 mg/L in a mono-culture to 172 mg/L [199]. Sometimes, the low substrate specificity of upstream enzymes may lead to unwanted conversions into downstream products [194]. In such cases, enzyme engineering can be used to modify the enzymes, or the pathway can be split and expressed in different strains. Horinouchi et al. [224] separated the genistein synthesis pathways and placed them in *E. coli* and yeast, achieving a higher production of 100 mg/L when mixed.

Similar to plant chassis, microbial heterologous synthesis faces challenges in compound transport, such as precursor uptake, material exchange between subcellular compartments, intermediate loss, and feedback inhibition due to insufficient final product secretion [225]. Zhao et al. [190] discovered that overexpressing OmpF, among the ten native transport proteins screened, significantly increased the production of the stilbene resveratrol, indicating that the screening of native transport proteins can be valuable, even though most of them may not be conducive to improving yield or cell growth when overexpressed. Anthocyanins are often stored in the central vacuole in plants, where the pH is lower than in the cytoplasm, which is more favorable for the stability of the products. Lowering the pH of the culture medium and maximizing the secretion of anthocyanins into the extracellular space can boost production. In a study that aimed to synthesize anthocyanin in *E. coli*, it was found that simultaneously knocking out the catechin efflux transporter tolC and overexpressing the product C3G efflux transporter yadH significantly promoted product synthesis, with tolC knockout having a greater impact [195].

Alkaloids, due to their high pKa, carry a positive charge during microbial fermentation, making them unable to cross the membrane by diffusion. Overexpressing a MATE transporter protein, AtDTX1, from *Arabidopsis* in the yeast cell membrane, increases the secretion of (S)-reticuline, an intermediate in benzylisoquinoline alkaloids (BIAs) biosynthesis, and enhances its production 11-fold [226]. Dastmalchi et al. [227] systematically screened and functionally identified a series of active transport proteins, BIA uptake permeases (BUPs), in opium poppy, which are involved in the intracellular transport of BIAs. Combining AtDTX1 and BUPs to regulate intermediate transport may make strategies involving the co-culture of different strains more promising for BIA production [201].

### 4.3. Fermentation Condition and Its Optimization

Once optimal recombinant strains have been obtained, the fermentation conditions become a critical last step in determining whether the production can be scaled up successfully. Specific conditions that affect strain performance include the composition and pH of the culture medium, environmental conditions, and the fermentation process itself. The composition of the culture medium can directly impact the yield of the recombinant strain. For instance, Liu et al. [207] found that peptones from different sources can affect the yield when synthesizing ajmalicine and sanguinarine in yeast. Additionally, the combination of components in the culture medium needs to be optimized in conjunction with the promoters used for pathway construction. For example, when using the Gal promoter, it is essential to pay attention to the ratio of galactose to other carbon sources and the timing of their addition. When using the HXT1 promoter, the concentration of glucose should be carefully managed. The pH of the culture medium plays a role in regulating aspects of the bioprocess, such as compound solubility, stability, and transport. Environmental conditions, such as aeration rate and temperature, can impact cell metabolism, growth, and product yield.

Similar to plant chassis, the timely removal or transfer of products is often used to enhance yield. For compounds with low polarity, like terpenes, two-phase culture methods that facilitate in situ product removal have been proven to significantly increase yield. For instance, adding diisononylphthalate to extract limonene has been shown to be effective [161].

## 5. Comparison and Prospects of Plant and Microbial Chassis

Assuming a standard of g/L as a high-yield chassis, a systematic analysis of cases for the production of plant-derived bioactive compounds in both plant and microbial chassis (Table 1 and Table 2) reveals the following:

1. In instances wherein plant chassis have succeeded and microbial chassis have not, they include the production of the diterpene paclitaxel, polyphenols such as anthocyanin, shikonin, rosmarinic acid, and the isoquinoline alkaloid berberine.
2. Microbial chassis have succeeded while plant chassis achieve lower yields for various monoterpenes, the sesquiterpene srtemisinic acid, the simple diterpene sclareol, and diterpene precursors like taxadiene and miltiradiene, the simple triterpene amyrin, linear tetraterpenes lycopene and β-carotene with little downstream tailoring enzyme modifications, polyphenolic upstream products like quercetin and (+)-catechin, and the isoquinoline alkaloid precursor (S)-reticuline.
3. Triterpenes like ginsenosides and polyphenolic compounds such as resveratrol can be produced at high yields in both plant and microbial chassis.

It is evident that microbial chassis are more efficient at producing structurally simple compounds or intermediates, while they face challenges in achieving high yields for complex bioactive compounds due to issues like interference with endogenous metabolism and low enzyme activity. For instance, even though the crucial isoquinoline alkaloid precursor (S)-reticuline can be produced at high yields in yeast (4.6 g/L) [203], it can only yield nM levels of downstream product morphine, even using (R,S)-norlaudanosoline as an externally added substrate [228]. Similarly, terpenoid indole alkaloids with complex synthesis pathways, such as vinblastine, face issues like promiscuous reactions, interactions between hosts and foreign substances, as well as challenges in expressing active enzymes. Plant chassis, especially those using natural hosts, excel in overcoming these challenges, often requiring simple fermentation condition optimizations to achieve high yields for certain bioactive components. However, due to the strict metabolic regulation and complex feedback mechanisms of plants, they may struggle to achieve high yields for even structurally simple products (Table 3).

**Figure 2:**
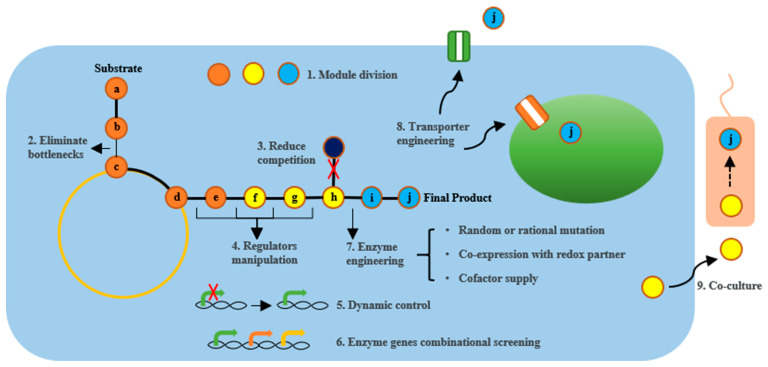
Schematic graph for strategies applied in plant secondary metabolites production in plant and microbial chassis. Substrate **a** undergoes a series of enzyme-catalyzed reactions to ultimately yield product **j** through intermediates **b**–**i**. Strategies for enhancing the yield of the final product include: **1.** Dividing the entire reaction pathway into several modules for individual optimization. **2.** Eliminating bottlenecks in the reaction pathway by overexpressing key enzyme genes. **3.** Knocking down or knocking out enzyme genes that catalyze intermediate products into other non-target substances. **4.** Enhancing overall reaction efficiency by adjusting the levels of regulators that simultaneously control multiple enzyme genes expression. **5.** Dynamically controlling enzyme gene expression using inducible promoters to reduce cellular burden or toxic side effects. **6.** Screening for the optimal combination of enzyme genes obtained from different species. **7.** Enhancing enzyme activity, stability, and adaptability to the host by amino acid site mutagenesis, co-expression of redox partners, cofactor engineering, etc. **8.** Storing target compounds in specific compartments by manipulating transport proteins. **9.** Splitting metabolic pathways into several parts, expressing them in different cell lines, and then obtaining the final product through co-cultivation.

From a strategic perspective, both plant and microbial platforms employ similar approaches, involving pathway construction or enhancement, overexpression of key enzyme genes, reduction in or elimination of competitive metabolic flux, enzyme engineering, and transport engineering (Figure 2). However, plants have complex, mostly unexplored metabolic pathways, lower genetic transformation efficiency, and a limited promoter library, making them less successful in various strategy aspects compared to microbes, such as dynamic control and precise expression regulation. Also, strategies for eliminating bottleneck steps have shown more excellent results in microbial platforms. Specifically, if the product yield significantly increases upon the addition of a specific intermediate, it indicates that the enzyme catalyzing the synthesis of that intermediate may have insufficient activity or the intermediate is being used by the endogenous pathway [144]. If no intermediates are available for use, one can consider using LC-MS to measure the abundance of metabolites throughout the entire pathway [203].

Although microbial chassis have the advantages and achievements mentioned above, they also face their own challenges. Since endogenous metabolic pathways are interconnected, optimal regulation strategies cannot be found without globally controlling the distribution of metabolic flux. For example, in the synthesis of tropine alkaloids using yeast, it was surprisingly found that removing leucine auxotrophy by expressing 3-isopropylmalate dehydrogenase significantly increased the conversion of hyoscyamine (85% decrease) to scopolamine (more than 3-fold increase) [229]. Current analysis of global metabolic flux can only be performed through somewhat unsatisfactory model construction and simulation [230], or more promisingly, using artificial intelligence [231,232]. However, they all rely on repeatable and reliable datasets, while standardized strains and operations are difficult to unify in the academic community. As mentioned above, transport engineering of secondary metabolites is a highly promising strategy. However, we currently have many gaps in the understanding of the functions and activity regulation mechanisms of plant secondary metabolite transporters and even microbial endogenous transporters. Therefore, this strategy has not yet reached its maximum potential.

In summary, in the current research progress, both microbial and plant platforms have seen successful cases. Due to the clearer metabolic pathways of microbes and the ease of genetic transformation and scale-up cultivation, they have achieved a series of remarkable accomplishments, especially eukaryotic yeasts, in the synthesis of various structurally uncomplicated compounds. In the future, if microbial platforms can successfully address global metabolic regulation, maintenance of heterologous enzyme activity, and issues related to metabolite transport, it is believed that even more success can be achieved. As our understanding of plants endogenous metabolism becomes clearer and more convenient genetic transformation methods and transcriptional regulatory elements are developed, the successful experiences on microbial platforms may guide plants to become a more versatile host.

## Figures and Tables

**Table 2:** Typical examples of plant secondary metabolites production in microbial chassis.

Compound	Product	Host	Engineering Strategy	Titer (mg/L)	Vessel	Ref.
Terpenoids						
Monoterpenes	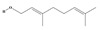 Geraniol	*E. coli*	Overexpression of heterotic geraniol synthase, GPP synthase, and mevalonate (MVA) pathway	2000	Fermentor	[158]
			Two-phase culture			
			Hydrolyzing geranyl acetate to geraniol by overexpression of acetylesterase			
		*S. cerevisiae*	Overexpression of isopentenyl diphosphate isomerase1 (*IDI1*) and truncated *HMGR*	1680	Fermentor	[159]
			Co-expression of the reverse fusion of truncated *GES-Erg20*^WW^ variant and another copy of *Erg20*^WW^ variant			
			Carbon restriction			
		*S. cerevisiae*	Deletion of OYE2 to reduce the endogenous metabolism of geraniol	1690	Fermentor	[160]
			Dynamic control of *ERG20* expression			
			Overexpression of *IDI1* and truncated *HMGR*			
	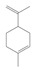 Limonene	*E. coli*	The two-liquid phase fed-batch setup	2700	Fermentor	[161]
			Limonene synthase from *Mentha spicata* and GPP synthase 2 from *Abies grandis*			
		*S. cerevisiae*	An orthogonal engineering by introducing truncated neryl diphosphate synthase from *Solanum lycopersicum* and limonene synthase from *Citrus lemon*	917.7	Flask	[162]
			Expression of *ERG20* regulated by the glucose-sensing promoter HXT1			
	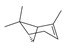 α-pinene	*E. coli*	Co-expressed *IspA* from *E. coli* and *Pt30* from *Pinus taeda*	970	Fermentor	[163]
			Heterologous MVA pathway and GPP synthase			
	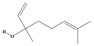 Linalool	*S. cerevisiae*	Directed evolution of linalool synthase	53.14	Flask	[164]
			Overexpression of the complete MVA pathway			
Sesquiterpenes	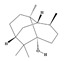 Patchoulol	*S. cerevisiae*	Fusion expression of FPP and patchoulol synthase	466.8	Flask	[165]
			Enhanced expression of the limiting genes of the MVA			
			Expression of squalene synthase driven by HXT1 promoter			
			Farnesol biosynthesis was inhibited			
		*S. cerevisiae*	Global metabolic engineering strategy	42.1	Flask	[166]
			Modulated expression of nine genes involved			
	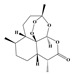 Artemisinic acid	*S. cerevisiae*	Down-regulation of ERG9 under CTR3 promoter in response to copper and reductase CRP1	25,000	Flask	[167]
			Additional introduction of CYB5, ADH1, and ALDH1			
		*S. cerevisiae*	ERG9 controlled with promoter Pmet3	2500	Fermentor	[168]
			Addition of methionine			
Diterpenes	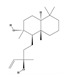 Sclareol	*S. cerevisiae*	Rewiring central metabolism for supplication of acetyl-CoA and NADPH	11,400	Fermentor	[169]
			Optimization of MVA pathway			
			Fusion of two diterpene synthase and maltose-binding protein			
			Knock-out regulatory factors			
		*E. coli*	Reconstruction of sclareol biosynthetic pathway	1500	Flask	[170]
			High-cell-density fermentation			
	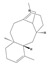 Taxadiene	*E. coli*	Optimally balanced expressions of up and downstream pathway modules	1020	Fermentor	[171]
			Two-phase cultivation			
		*S. cerevisiae*	Fusion of GGPPS and taxadiene synthase	184.2	Fermentor	[172]
	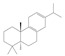 Miltiradiene	*S. cerevisiae*	Overexpression of MVA pathway genes	3500	Fermentor	[173]
			Downregulation of transcription factor ROS1 and competing enzyme ERG9			
			Fusion of two Class I and Class II terpene synthases			
	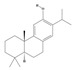 Ferruginol	*S. cerevisiae*	Fusion of GGPP synthase and FPS; fusion of copalyl diphosphate synthase and kaurene synthase-like	10.5	Flask	[174]
			Co-expression with plant CPR			
Triterpenes	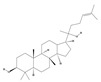 Ginsenosides	*S. cerevisiae*	Semi-rational design of UGT51	300	Fermentor	[175]
			Preventing Rh2 degradation and increasing UDP-glucose precursor supply			
		*S. cerevisiae*	Modular engineering of the MVA and optimization of P450 expression levels	2250	Fermentor	[176]
			Increasing the copy number and expression level of UGTPg45 and direct evolution by random mutation			
	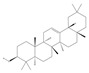 Amyrin	*S. cerevisiae*	Introducing efficient cytochrome P450s and pairing their reduction systems	108.1	Fermentor	[177]
			By increasing the copy number of Uni25647 and pairing CPRs			
		*S. cerevisiae*	Enzyme engineering of amyrin synthase	1108	Fermentor	[178]
			Overexpression of MVA pathway and diacylglycerol acyltransferase			
	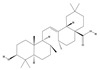 Oleanolic acid	*S. cerevisiae*	Screening for better CPR	606.9	Fermentor	[179]
			Knock-out galactokinase (GAL1) and negative transcriptional regulator (GAL80)			
	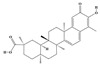 Precursors of celastrol	*S. cerevisiae*	Increase endogenous cytosolic MVA pathway	15.3	Flask	[180]
Tetraterpenes	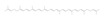 Lycopene	*S. cerevisiae*	Adjusting the copy number of three key genes, knocking-out endogenous bypass genes	3280	Flask	[181]
			Increasing the supply of the precursor acetyl-CoA, balancing NADPH utilization			
			Using GAL-inducible system			
		*E. coli*	Modulating three genes to increase ATP and NADPH supply	3520	Fermentor	[182]
			Modulating pathway gene expressions by screening RBS library			
	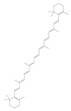 β-carotene	*S. cerevisiae*	Over-expression of *HMGR*	6.29 mg/g DW	Flask	[183]
			Addition of ergosterol biosynthesis inhibitors			
		*S. cerevisiae*	Reconstructing controllable multi-gene pathways by employing the GAL regulatory system	20.79 mg/g DW	Flask	[184]
			Optimized by repeatedly using GAL10-GAL1 bidirectional promoters with high efficiency			
	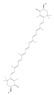 Astaxanthin	*S. cerevisiae*	Introducing the GAL regulation system	235	Fermentor	[185]
			Adopting temperature as an input signal			
		*S. cerevisiae*	Physical mutagenesis by ARTP and adaptive evolution driven by H_2_O_2_	404.78	Fermentor	[186]
Phenols						
Isoflavones	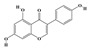 Genistein	*E. coli*	Screening for better enzymes	35	Flask	[187]
			Translational fusion of RcIFS and OsCPR			
		*S. cerevisiae*	Screening for CHS	31.02	Flask	[188]
			Enhancement of precursor pathway and expression of feedback-resistant enzyme genes			
			Optimization of enzyme subcellular localizations			
	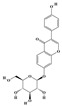 Daidzin	*S. cerevisiae*	Screening for pathway genes	73.2	Flask	[189]
			Screening and overexpression of CPR partners			
			Increase co-factors supply			
			Dynamic control of p-coumaric acid supply			
			Enzyme fusion			
Stilbenoids	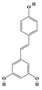 Resveratrol	*E. coli*	Screening for pathway genes	238.71	Flask	[190]
			Antisense inhibition of competing pathway to increase malonyl-CoA supply			
			Transport engineering for resveratrol secretion			
			Expression of chaperones to aid enzyme folding			
		*S. cerevisiae*	Enhance precursor pathway and expression of feedback-resistant enzyme genes	4100	Fermentor	[191]
Flavanols	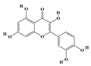 Quercetin	*S. cerevisiae*	Engineered a delphinidin-overproducing *S.cerevisiae*-*S. cerevisiae* co-culture	154.2	Fermentor	[192]
	Quercetin	*S. cerevisiae*	Combinational screening for downstream enzyme genes	930	Fermentor	[193]
	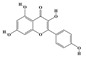 Kaempferol			956		
			Increase gene copy numbers in genome			
			Enhance precursor pathway and expression of feedback-resistant enzyme genes			
Anthocyanins	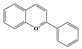 Anthocyanin	*S. cerevisiae*	Screening pathway enzymes from different plants	5.4 (total anthocyanins)	Flask	[194]
	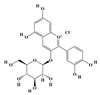 cyanidin 3-O-glucoside	*E. coli*	Enhancing substrate and precursor availability	350 (from (+)-catechin)		[195]
			Balancing gene expression			
			Manipulate transportation by tolC knockout and yadH overexpression			
Proanthocyanidin	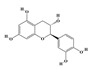 (+)-catechin	*E. coli*	Combinatorial screening pathway genes	911 (from eriodictyol)	Flask	[196]
			Improving the availability of NADPH			
Naphthoquinone	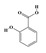 3-geranyl-4-hydroxybenzoate acid	*S. cerevisiae*	Increase GPP supply by increasing HMG1 and Erg20(K197G)	179.29 (from hydroxybenzoate acid)	Flask	[197]
	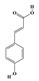 p-Coumaric acid	*S. cerevisiae*	Screening for p-hydroxybenzoate:geranyltransferase	1890	Fermentor	[198]
			Overexpression of feedback inhibition-resistant DAHP synthase and chorismate mutase			
			Knockout aromatic product pathway genes ARO10 (phenylpyruvate decarboxylase) and PDC5 (pyruvate decarboxylase)			
Hydroxycinnamic acids	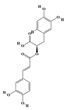 Rosmarinic acid	*E. coli*	Establishment of partial pathway in different strains and individual optimization and combination of strains	172	Flask	[199]
Alkaloids						
Tropane alkaloids	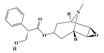 Scopolamine	*S. cerevisiae*	Transporter engineering by disrupting TPO5 and overexpression vacuolar tropine importers and two exporters	0.172	Flask	[200]
			Overexpression NADPH regeneration-related genes			
			Tryptophan prototrophy			
Isoquinoline alkaloids	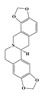 Stylopine	*E. coli and P. pastoris*	Stylopine and reticuline module synthesized in *P. pastoris* and *E. coli*, respectively	1.615	Flask	[201]
			Inoculation ratio and medium were optimized			
	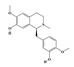 (S)-reticuline	*E. coli*	Introducing two feedback-resistant enzymes	307	Flask	[202]
			Establishment of an additional heterologous pathway			
		*S. cerevisiae*	Knock-out of competing oxidoreductases consuming 4-hydroxyphenylacetaldehyde	4600	Fermentor	[203]
			Increase 4-hydroxyphenylpyruvate and dopamine supply			
			Screening and engineering norcoclaurine synthase			
	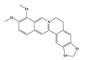 Berberine	*S. cerevisiae*	Knockout competing oxidoreductases consuming 4-hydroxyphenylacetaldehyde	1.08	Fermentor	[204]
			Enhancement of the supply of tyrosine			
Terpenoid indole alkaloid	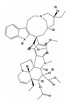 Vinblastine	*S. cerevisiae*	Screening for better enzymes	0.4	Flask	[205]
			Downregulation of competing enzyme genes			
			Co-expression with CPRs			
	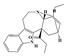 Catharanthine	*Pichia pastoris*	Three modules integrated in stable genome integration sites	2.57	Fermentor	[206]
			Optimize copy number of pathway genes			
			Increase in cofactor, S-adenosyl-l-methionine supply, and co-expression with CPR genes			
			Induction medium optimization			
	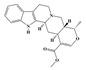 Ajmalicine	*S. cerevisiae*	Stable integration in the genome	61.4	Flask	[207]
			Increase precursor supply by increasing gene copies and knocking-out competing pathway genes			
			Co-expression with CPRs			

**Table 3:** Advantages and disadvantages of plant and microbial chassis.

Host	Advantages	Disadvantages
*Plant*	The natural host has prepared cofactors and precursors; Organelles and higher-order membrane structures allow for the storage of intermediates and products; Rich redox partners that are better matched with P450 enzymes.	Complex endogenous metabolic pathways that interfere with the target pathway; Strict metabolic regulation, including potential unknown regulators; Transgene expressions are prone to silencing; Limited gene expression elements and unclear driving force of promoters; Slow growth rates; Demanding fermentation conditions and bioreactors and challenging to achieve high-density fermentation.
*Microbes*	Relatively clear endogenous metabolic pathways and regulatory mechanisms; Convenient and rapid genetic manipulation; Abundance of standardized expression elements; Fast growth rates; Easy achievement of large-scale and high-density cultivation.	Lack of advanced membrane structures and organelles; Activity of exogenous enzymes may be hindered; Additional provision of redox partners matching P450 enzymes and increased cofactor supply are required.

## Data Availability

All data are contained within the article.
